# The ‘Unicorn’ Dinosaur That Wasn’t: A New Reconstruction of the Crest of *Tsintaosaurus* and the Early Evolution of the Lambeosaurine Crest and Rostrum

**DOI:** 10.1371/journal.pone.0082268

**Published:** 2013-11-22

**Authors:** Albert Prieto-Márquez, Jonathan R. Wagner

**Affiliations:** 1 Bayerische Staatssammlung für Paläontologie und Geologie, Munich, Germany; 2 Department of Geography, Texas State University-San Marcos, San Marcos, Texas, United States of America; Monash University, Australia

## Abstract

The lambeosaurine *Tsintaosaurus spinorhinus* has traditionally been reconstructed with an elevated, hollow, spike-like crest composed entirely of the nasal bones, although this has been disputed. Here, we provide a new reconstruction of the skull of this species based on reexamination and reinterpretation of the morphology and articular relationships of the type and Paratype skulls and a fragmentary crest. We confirm the presence of a supracranial crest composed of the elevated nasal bones, but also including the premaxillae. We hypothesize that the crest is a tall, lobate, hollow structure that projects dorsally and slightly caudally a distance greater than the height of the skull along the quadrate. In our reconstruction, the nasal passage passes through the crest, but enters the skull rostral to the tubular process of the nasals, not through it. *Tsintaosaurus spinorhinus* is rediagnosed on the basis of a suite of cranial autapomorphies including a circumnarial fossa subdivided into three accessory fossae, prefrontal with ascending rostral process and lateral flange, nasals fused sagittally to form elongate tubular process that rises dorsally from skull roof, each nasal being expanded rostrocaudally into a rhomboid distal process, and medial processes of premaxillae at the summit of the cranial crest inserted between rhomboid processes of nasals. *Tsintaosaurus spinorhinus* lacks characters that are present in more derived lambeosaurines (parasaurolophins and lambeosaurins), such as rotation of the caudal margin of the crest to an acute angle with the skull roof, lateral processes of the nasals that enclose part of the intracranial cavity and participate in the formation of the walls of the common median chamber, and a smooth narial fossa lacking ridges and accessory fossae. We hypothesize that ancestrally the rostrum of lambeosaurines may have been more similar to that in Saurolophinae, and became subsequently reduced in complexity during evolution of the group.

## Introduction

Lambeosaurine hadrosaurids are the most anatomically derived ornithopod dinosaurs [1]. They are unique among ornithischians in having a caudodorsally extended nasal passage enshrouded by hollow supracranial crests of a wide variety of forms and sizes [2,3]. Lambeosaurines have been recorded in the Late Cretaceous of Eurasia and North America [4], particularly in the Campanian-Maastrichtian of the northwestern United States and southern Canada [5], and tentatively in the Early Maastrichtian of Argentina [6,7]. 

An important question in the evolutionary history of lambeosaurines is how these animals evolved such a derived configuration of the facial complex in connection with caudodorsal migration of the nasal passage. Of particular interest are the anatomical transformations that took place during the early evolution of lambeosaurines from their hadrosaurid ancestors that dramatically affected the morphology the main crest-forming bones, namely, the premaxilla and nasal. A logical approach to exploring this question is to investigate morphological attributes and relationships of these bones in lambeosaurines whose phylogenetic positions bracket the base of the phylogeny. These characters can then be optimized on the phylogenetic tree in order to reconstruct the sequence of character changes along early branches. According to the latest phylogenetic hypotheses [8–14], the relevant lambeosaurine species are *Aralosaurus tuberiferus* [15], *Jaxartosaurus aralensis* [16], *Tsintaosaurus spinorhinus* [17], and *Pararhabdodon isonensis* [18]. Among these, only *T. spinorhinus* preserves complete nasals and partial premaxillae. Only the caudal-most extent of the nasal is available for *A. tuberiferus* [15,19], and neither this bone nor the premaxilla is preserved in *J. aralensis* [1,16] and *P. isonensis* [20,21].

*Tsintaosaurus spinorhinus* was originally described by C. C. Young in 1958 from partially articulated cranial and postcranial materials collected from a bonebed in the Campanian (Upper Cretaceous) Jingangkou Formation of Shandong, China [22]. This dinosaur is remarkable for purportedly sporting a long, hollow tubular structure formed by the nasals projecting dorsally and slightly rostrally from the rostrodorsal region of the skull. Some authors have questioned whether this structure represents a hollow crest. Weishampel and Horner [23] were unconvinced that the crest was hollow and regarded this taxon a mixture of saurolophine (premaxilla, skull roof, and neurocranium) and lambeosaurine (maxilla, quadrate, and most dentaries) material. Taquet [24] suggested that the upright orientation of the nasals was caused by caudodorsal *post-mortem* distortion of these bones. He proposed that the nasals should be reconstructed in a rostroventral orientation as in saurolophine hadrosaurids. However, Buffetaut and Tong-Buffetaut [25] provided osteological evidence supporting the dorsal projection of the nasal crest as originally described and figured by Young [17]. These authors observed that the rostral region of the frontals, near the articulation with the nasals, is rostrodorsally curved and contributes to the base of the crest. Likewise, they also noted similar morphology in the Paratype skull, not considered by previous authors, including dorsally deflected frontals and a prefrontal with a rostral process that is medially and dorsally projected and buttresses the base of the nasal crest.

Among the remains recovered with the nominal hypodigm of *Tsintaosaurus spinorhinus*, Young [17] also recovered a partial premaxilla and nasal that he attributed to an indeterminate lambeosaurine. Here we present evidence that this element is in fact the partial medial premaxillary process of *T. spinorhinus*. We provide a revised reconstruction of the facial complex and crest in this species based on new information from this premaxilla and nasal, as well as reexamination of the type material. The new anatomical observations presented in this study lead to a revised diagnosis of *T. spinorhinus*. The resulting model of the cranial anatomy of *T. spinorhinus* is placed in phylogenetic context, and used to draw insights into character evolution of the facial complex among lambeosaurines.

## Results

### Systematic Paleontology

**Figure 1:**
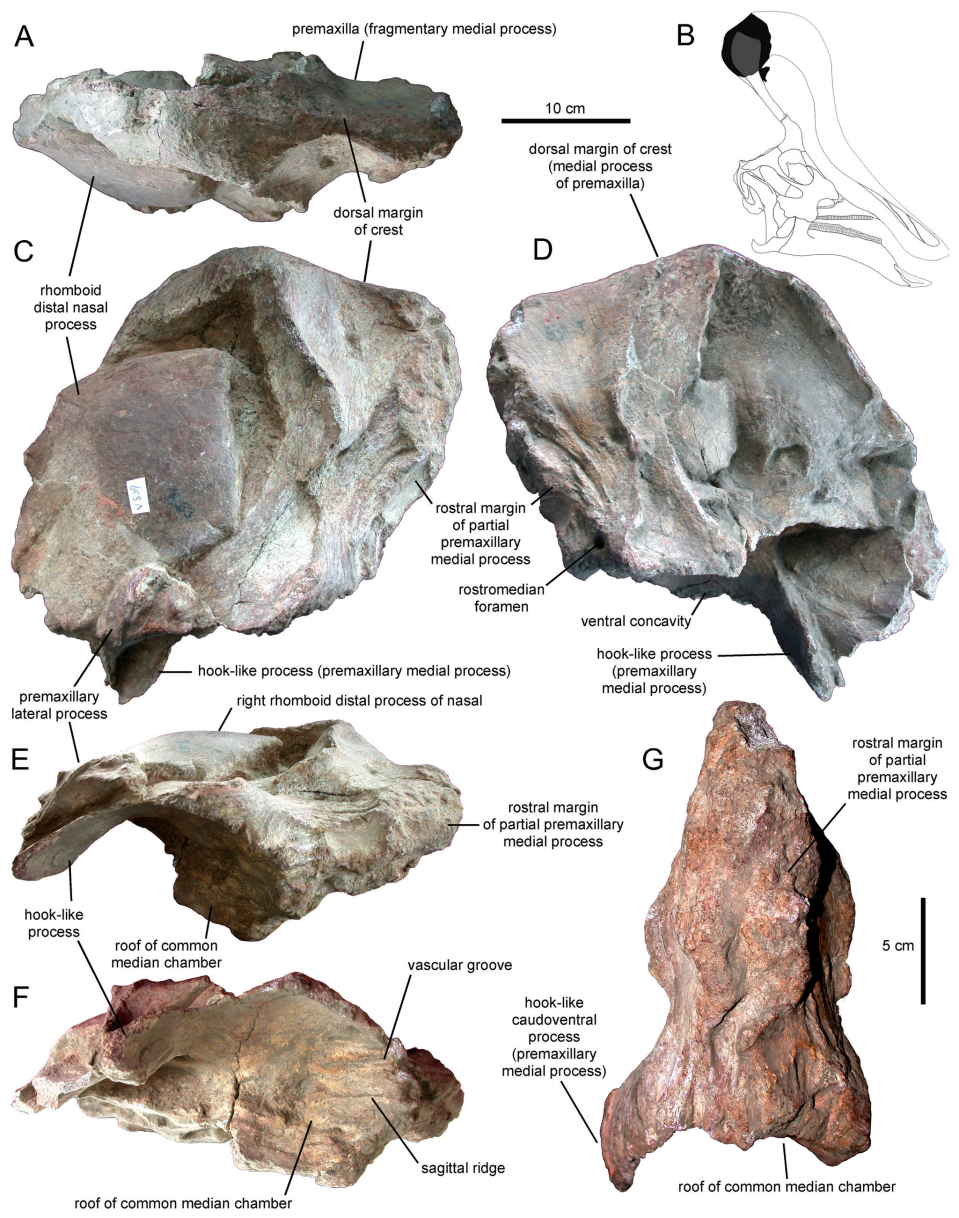
Partial premaxillae and partial right nasal of *Tsintaosaurus spinorhinus* (IVPP V829). Specimen in (A) dorsal, (C) right lateral, (D) left lateral, (E) ventral, (F) rostroventral, and (G) rostral views. B. Insert of our reconstruction of the skull of *T. spinorhinus* showing the position of the prefrontal (solid black).

**Figure 2:**
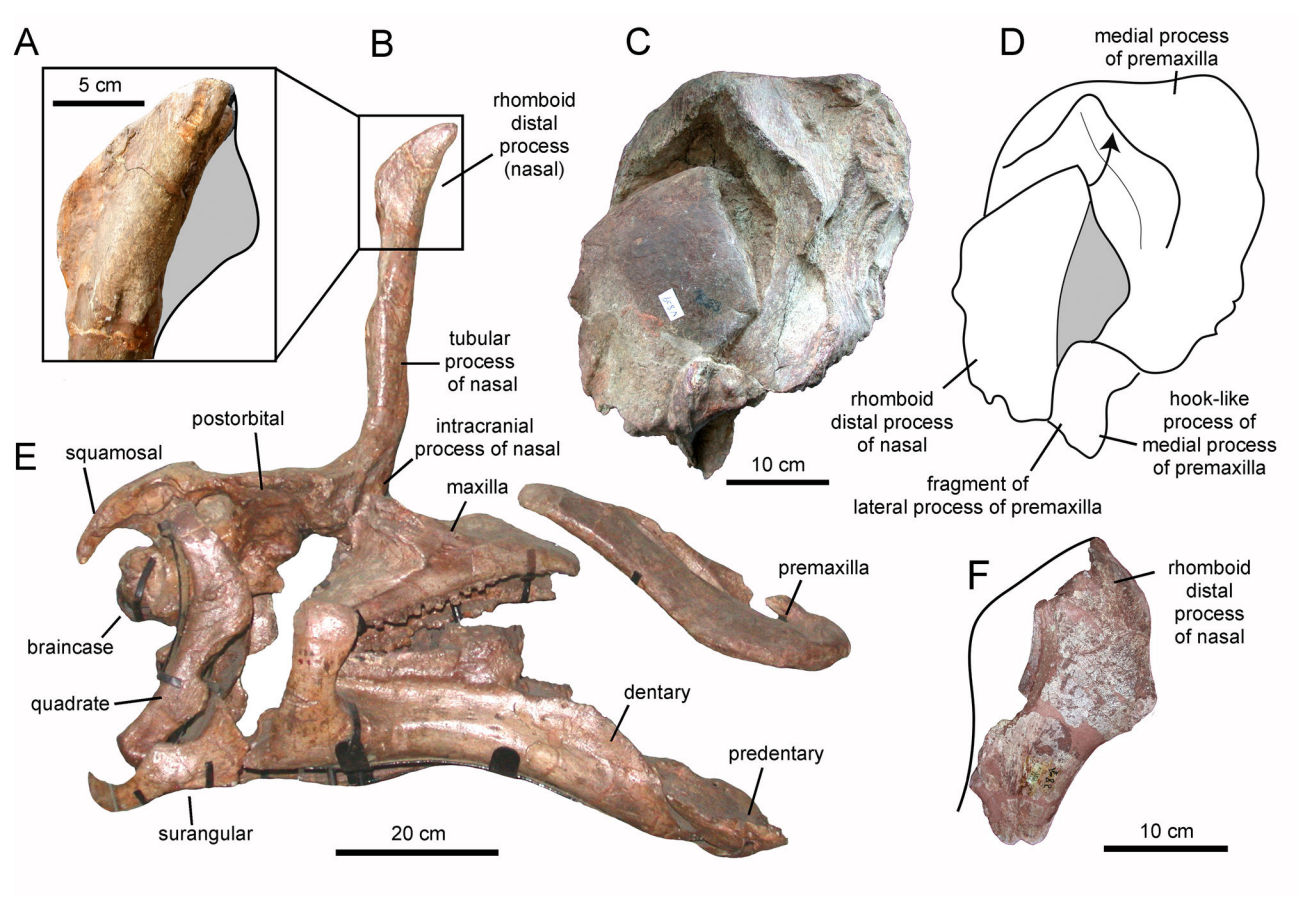
Rhomboid distal nasal process of the crest of *Tsintaosaurus spinorhinus*. A. Partial distal nasal process of IVPP V725 in right lateral view. B. Lateral view of composite skull (reversed). C. partial premaxillonasal complex of IVPP V829 in right lateral view. D. Line drawing of (C) showing nasal-premaxilla articulation, and the displacement (arrow) experienced by the distal nasal process relative to its articular position. E. Mounted holotype skull (IVPP V725) of *T. spinorhinus* in left lateral view (reversed). F. Partial right distal nasal process (although catalogued as IVPP V725, this element corresponds to a different specimen than the type; reversed).

**Figure 3:**
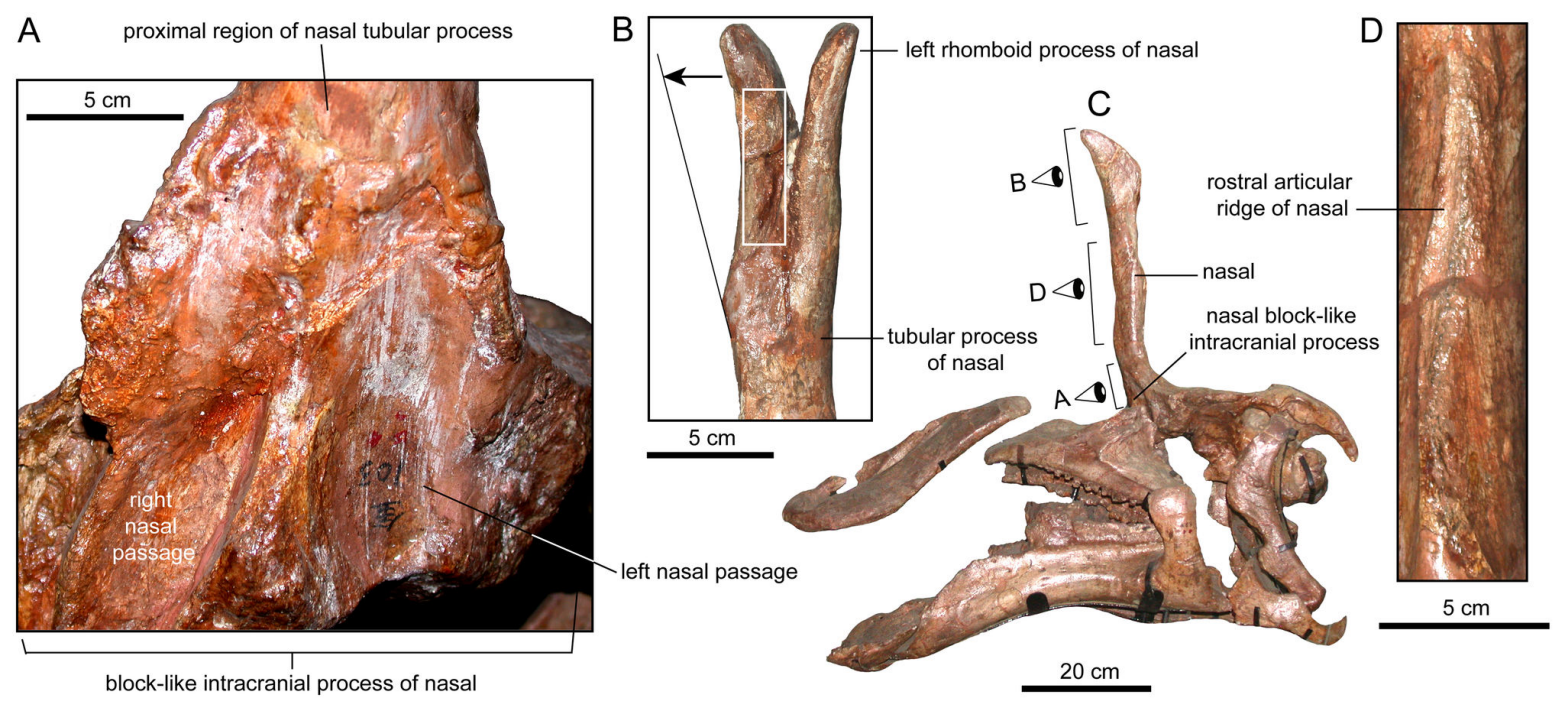
Nasal anatomy of *Tsintaosaurus spinorhinus* (IVPP V725). A. Paired depressions for the nasal passage in rostroventral view. B. Distal rhomboid processes of the nasal of IVPP V725 in rostroventral view, showing areas of missing bone (white rectangle) and medial post-depositional displacement of the right process (double arrow). C. Composite skull in left lateral view. D. Rostral view of the sagittal ridge present on the rostral surface of the tubular processes of the fused nasals, interpreted here as the articular surface for the lateral process of the premaxilla.

**Figure 4:**
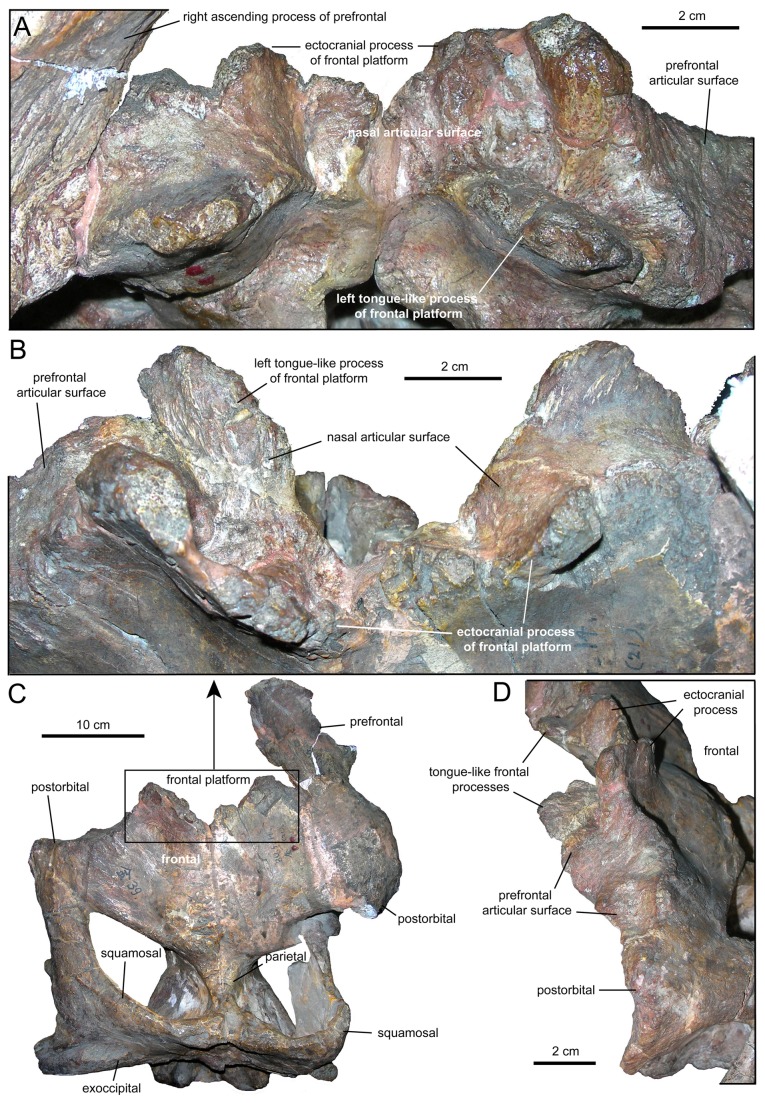
Nasofrontal articulation of *Tsintaosaurus spinorhinus*. Paratype partial skull, IVPP 818. A. Rostral view. B. Rostrodorsal view. C. Dorsal view; note that the nasofrontal articulation is covered by the ecotcranial surface of the frontal. D. Left rostrodorsolateral view.

**Figure 5:**
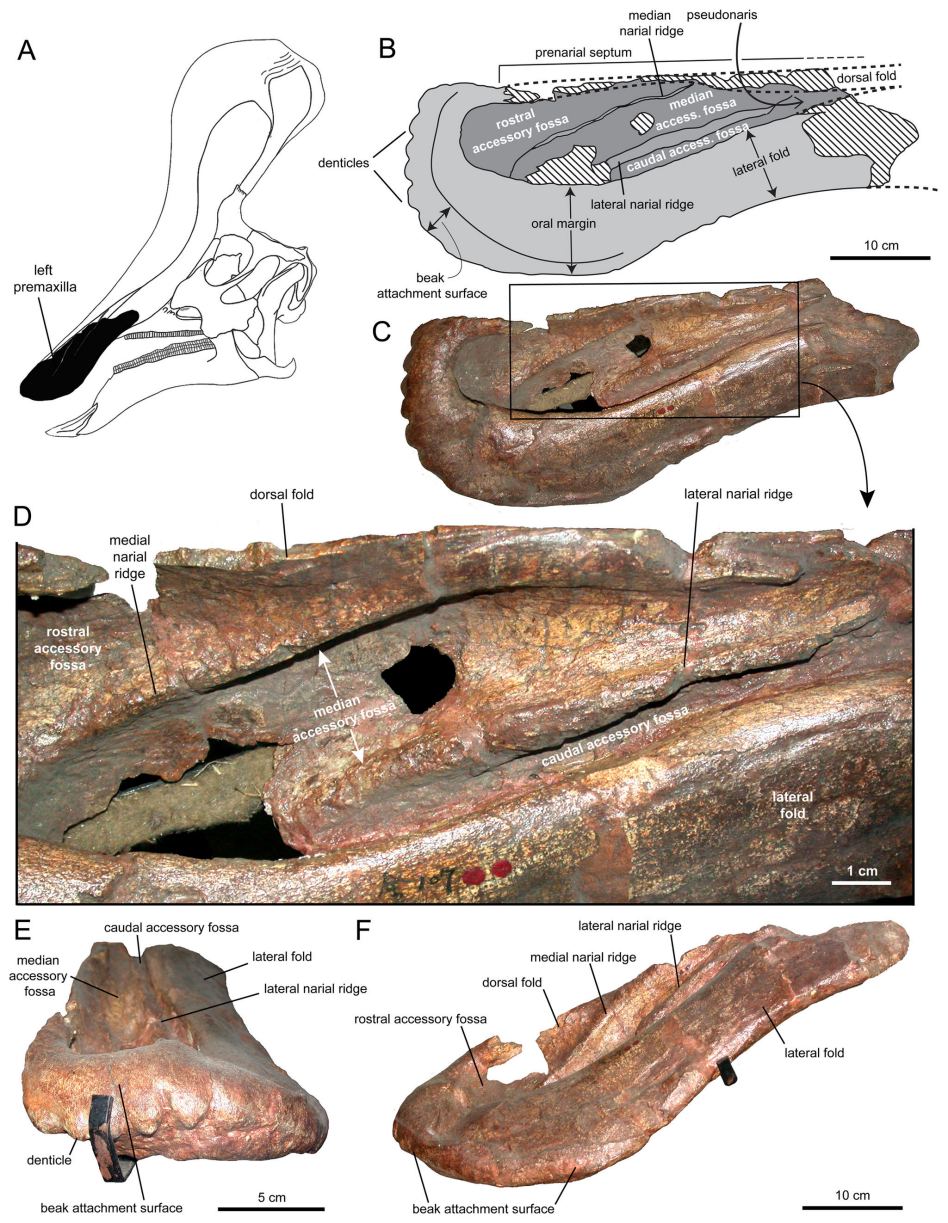
Left premaxilla of *Tsintaosaurus spinorhinus* (IVPP K107). Premaxilla in (A) rostrodorsal and (C) rostral views. B. Insert of our reconstruction of the skull of *T. spinorhinus* showing the position of the prefrontal (solid black).

**Figure 6:**
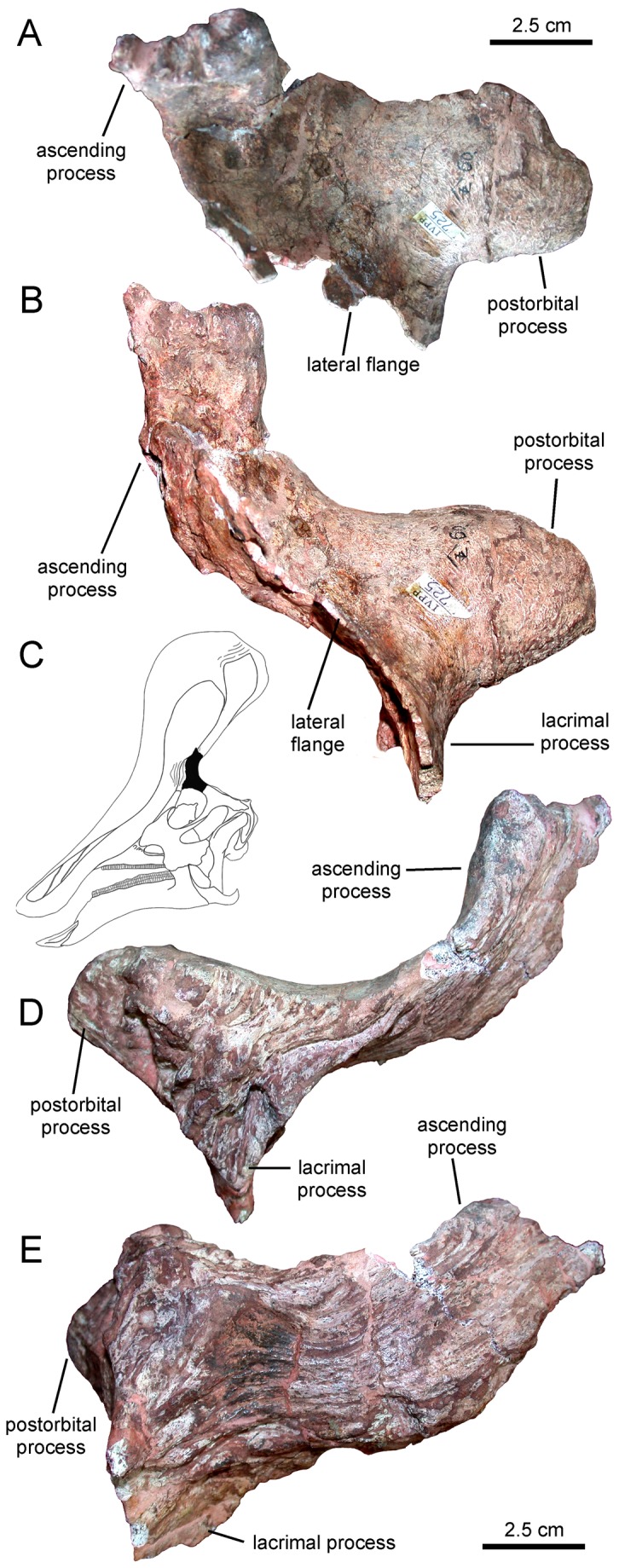
Left prefrontal of *Tsintaosaurus spinorhinus* (IVPP V725). Prefrontal in (A) dorsal, (B) lateral, (D) medial, and (E) medioventral views. C. Insert of our reconstruction of the skull of *T. spinorhinus* showing the position of the prefrontal (solid black).

**Figure 7:**
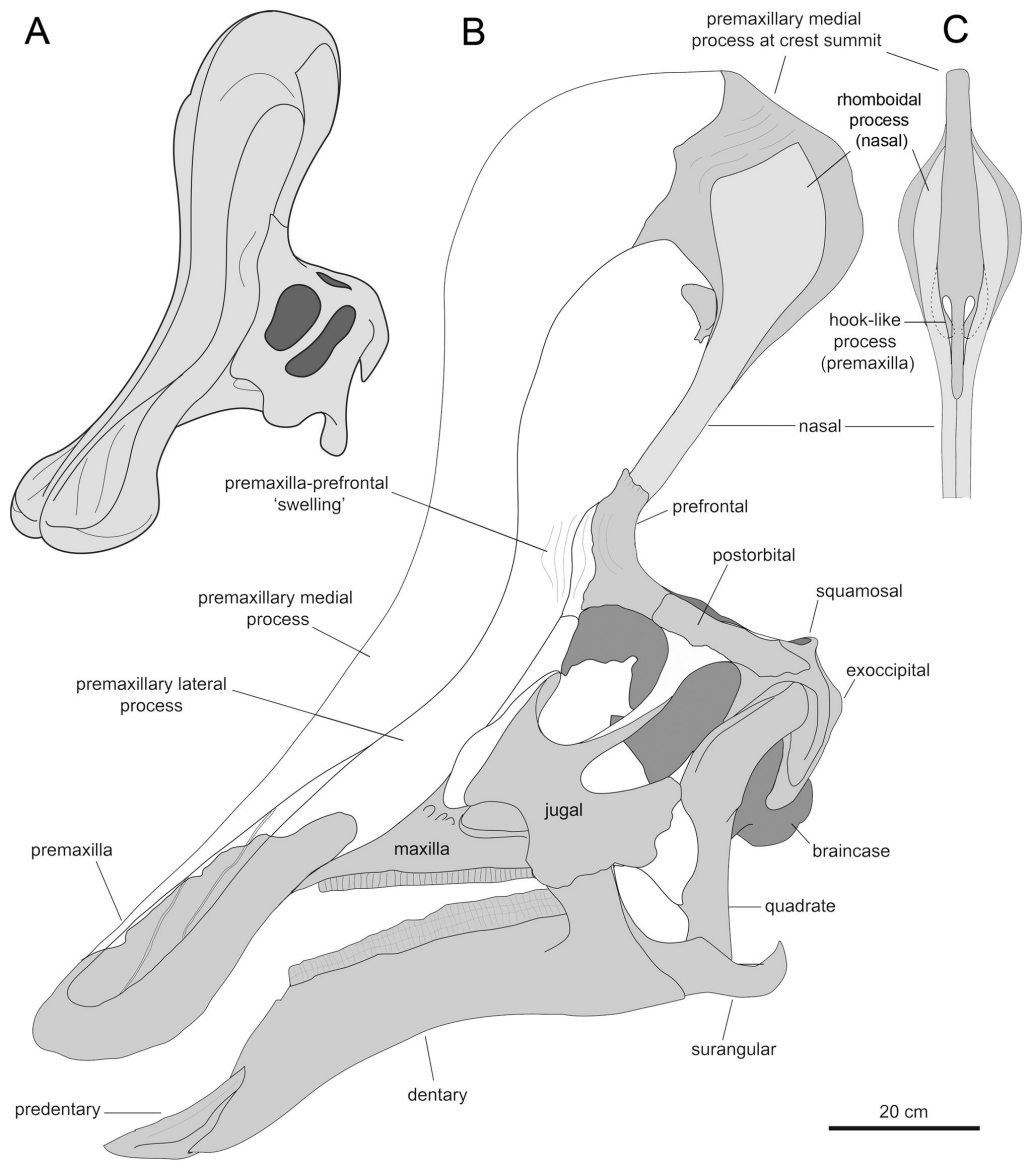
Anatomical model of the cranium and supracranial crest of *Tsintaosaurus spinorhinus*. A. Model of the skull in rostrolateral view. B. Lateral view of the model based on IVPP V723, V725, V829, and K107. C. Caudal view of the dorsal region of the crest. In B and C, grey areas indicate the available bones; white areas are missing bones and hypothesized reconstructions of missing parts of bones.

**Figure 8:**
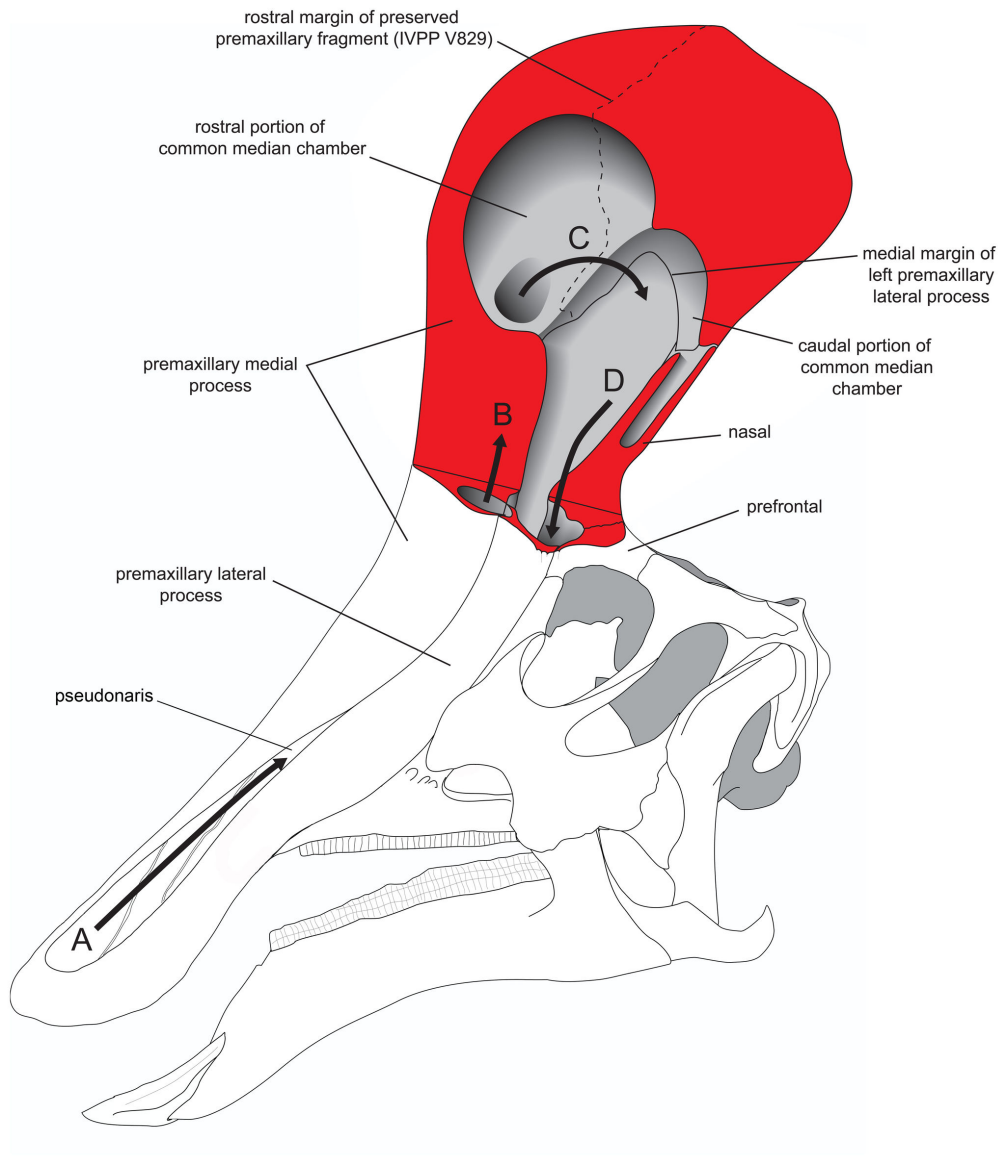
Model of internal crest anatomy of *Tsintaosaurus spinorhinus*. Based on the model in Fig. 7. Red indicates cut bone; arrows indicate hypothetical direction of airflow. A. Air enters the nasal passage through the left pseudonaris. B. Air passes dorsally into the crest from the left pseudonaris. C. Air from the right pseudonaris enters the common median chamber and passes caudally. D. Air from the left pseudonaris passes ventrally along the caudal margin of the crest into the cranial cavity.

Dinosauria Owen, 1842 [26] (sensu Butler et al., 2008 [27])
Ornithischia Seeley, 1887 [28]
Ornithopoda Marsh, 1881 [29]
Hadrosauridae Cope, 1870 [30] (sensu Prieto-Márquez, 2010 [13])
Lambeosaurinae Parks, 1923 [31] (sensu Prieto-Márquez, 2010 [13])
Tsintaosaurini Prieto-Márquez et al., 2013 [32]
Tsintaosaurus Young, 1958 [17]
Tsintaosaurus spinorhinus Young, 1958 [17]
(Figures 1, 2, 3, 4, 5, 6, 7, 8)

#### Holotype

IVPP (Institute of Vertebrate Paleontology and Paleoanthropology, Beijing, China) V725, partial articulated skull composed of frontals, braincase, parietal, squamosals, postorbitals, left prefrontal, and nasals. Young [17] included in IVPP V725 various axial and appendicular elements that may or may not correspond to individuals other than the braincase exemplar. These bones include left and right partial distal nasal processes, a series of cervical, dorsal, and caudal vertebrae, a complete sacrum, a left scapula and coracoid, left and right sternal plates, left and right humeri, left ilium, right and partial left ischia, partial left pubis, right and partial left femora, partial left and right fibulae, and distal left tibia ([17]: text figs 15—18, 21—23, 26—28, 30, and 31 respectively).

#### Paratype

IVPP V818, partial articulated cranium composed of frontals, braincase, parietal, squamosals, postorbitals, and left prefrontal ([17]:fig. 4).

#### Referred material

IVPP V723, predentary, and left and right dentaries; V727, left and right humeri; V728, sacrum, left and right humeri, right ilium, right ischium, right pubis, and left femur; V729, sacrum and left humerus; V768, isolated dentary tooth crowns; V829, articulated dorsal fragment composed of rhomboid, distal nasal processes and dorsal region of premaxillae; and V830, partial left and nearly complete right jugal; also, IVPP field numbers K24, partial predentary; K63, predentary; K28, left quadrate and right maxilla; K45, left maxilla; K63, left and right dentaries; K68, nearly complete left quadrate; K69, left ulna; K70, right coracoid and scapula; K82, right ulna; K86, left radius; K93, left maxilla; K96, right coracoid and scapulae; K97, right surangular; K107, left rostral region of premaxilla; K141, predentary; K149, left surangular; K150, left surangular; K155, dentary tooth; K156, several metacarpal and manual phalanges from various individuals; and K197, left and partial right dentaries. Additional material, referred to *Tanius* by Young [17], may or may not belong to this species, and we consider *Tanius chingkankouensis* Young, 1958 a nomen dubium pending further revision.

#### Occurrence

Lower Campanian [33] Jingangkou Formation, Wangshi Group, near the village of Chingkankou, 20 km south of the city of Laiyang, Shandong Province, eastern China [17,22].

#### Emended diagnosis

Lambeosaurine hadrosaurid possessing the following autapomorphies: oral margin of premaxilla with widely arcuate rostroventral corner and thick oral margin laterally broader than rostral-most extent of circumnarial fossa; cirumnarial fossa of premaxilla obliquely divided longitudinally by two rostrolaterally-oriented ridges; tall lobate supraorbital crest composed of nasals and premaxillae that projects dorsally and slightly caudally a distance greater than the height of skull along quadrate; nasals fused sagittally to form elongate tubular process rising dorsally from skull roof; nasals form block-like intracranial process rostral to braincase; nasal articulates with frontal by clipping between tongue-like ventral process and overhanging ectocranial process of frontal; distal end of nasal expanded rostrocaudally into rhomboid process; medial processes of premaxillae at summit of cranial crest, inserted between rhomboid processes of nasals; mediolaterally expanded caudal half of medial processes of premaxillae at summit of cranial crest, with lateral surface containing rhomboid facet for articulation with distal nasal process; medial process of premaxilla with hook-like intracranial process curving caudoventrally and slightly medially, subdividing common median chamber; prefrontal with ascending rostral process that projects dorsally while twisting medially to face and overlap proximal region of tubular process of nasal and part of lateral process of premaxilla; prefrontal with flange extending laterally from ventral region of lacrimal process to ventral region of ascending process, articulating with lateral process of premaxilla to form swelling on lateral preorbital region of skull; lateral and ventral surfaces of prefrontal bearing deep dorsoventrally-oriented grooves; and supratemporal fenestra wider mediolaterally than rostrocaudally. Additionally, *T. spinorhinus* differs from all other hadrosaurids, except *Pararhabdodon isonensis*, in possessing maxilla with elevation of jugal facet such that ventralmost extent is well above the level of the ectopterygoid ridge, with acute embayment ventral to jugal process between jugal facet and ectopterygoid ridge; it differs from *P. isonensis* in having a rostrocaudally narrower rostrodorsal region of the maxilla.

### Facial Morphology of *Tsintaosaurus spinorhinus*

#### Nasal

Young figured and briefly described a fragmentary specimen, IVPP V829, as part of the nasal and premaxilla from the crest of an indeterminate lambeosaurine ([17]: text fig. 40). This referral was later supported by Weishampel and Horner [23]. According to Young [17], the specimen was found in the same gully where the type and hypodigm of *Tsintaosaurus spinorhinus* were excavated. Overall, the fragment is subrectangular in lateral profile and mediolaterally compressed, containing the sagittal plane of the skull (Figure 1). On the best-preserved right side two elements are recognizable: a rhomboid mediolaterally compressed fragment and a surrounding bony mass (Figure 1B). We concur with Young [17] in that the rhomboid element is the nasal and the surrounding bone the premaxilla, and we further suggest that both elements belong to *Tsintaosaurus spinorhinus*. This identification is based on the fact that the rhomboid profile of the nasal in IVPP V829 (Figures 1B and 2C) is very similar to the fan-shaped and rostrocaudally expanded lateral profile of the distal process of the IVPP V725 nasal (Figure 2A and B). The rostroventral margin of the distal nasal process of IVPP V725 appears straight in lateral profile (Figure 2A); however, this straight profile results from breakage of the process. Close examination of the ventral surface of this margin reveals a flat fractured surface where bone is missing (Figure 3B, white rectangle). Thus, we interpret the distal nasal process of IVPP V725 as the incomplete caudodorsal half of the rhomboid structure preserved in IVPP V829. 

In IVPP V829 the distal process of the nasal fits tightly into a deep fossa on the lateral surface of the premaxilla (Figure 2D). The margins of the fossa mirror the rhomboid outline of the distal nasal process. One of the margins of the distal process is gently convex, whereas the other border is slightly sinuous. Given that the caudal margin of the distal process in IVPP V725 is gently convex, we consider the convex margin in the distal nasal process of IVPP V829 to correspond to the caudal margin and the gently sinuous border as the rostral edge of the bone. The rhomboid distal nasal process and its articulation with the premaxilla are well preserved on the right side of IVPP V829, but heavily eroded on the left side (where it is uncertain whether fragments of the nasal process remain; Figure 1D).

In addition to the type skull and IVPP V829, there are two other bone fragments morphologically consistent with the rhomboid nasal process and, thus, referable to *T. spinorhinus*. Both of them bear the IVPP V725 catalogue number; however, they must be part of specimens other than the type because, as shown above, the latter already bears both distal nasal processes. One of them (Figure 2F) preserves slightly more than the rostral half of the left distal process. The other piece is very fragmentary and appears to represent only the caudal half of a right distal process. Other putative nasals tentatively referred to *T. spinorhinus* by Young ([17]: text fig. 42) are less certain: IVPP K140 is almost certainly a first ceratobranchial, and IVPP K119 may be as well.

In *Tsintaosaurus spinorhinus* each rhomboid process of the nasal diverges laterally from the sagittal plane of the tubular process (Figure 3B). Thus, the elongate spike-like structure formed by the fused tubular processes of nasals (which are hollow according to Young [17]: text fig. 2) opens distally to accommodate the intervening premaxillae; each rhomboid process articulates on the lateral surface of each premaxilla as seen in Figure 1B. The separation between the rhomboid processes is present in IVPP V725: near the base of these processes, the nasals bifurcate and each element deviates laterally from the sagittal plane of the skull (Figure 3B). At first glance, this separation may appear too narrow in IVPP V725 to allow insertion of the premaxillae in between the rhomboid processes of the nasals at the sagittal plane of the skull. However, the right nasal process of IVPP V725 is cracked and post-depositionally displaced medially so that it appears closer to the left nasal than it was prior to deformation (Figure 3B, arrow). 

Ventral to the rhomboid processes, the nasals of IVPP V725 form their distinctive tubular structure as described by Young [17]. That the nasals might be hollow is not itself unusual; typically among hadrosaurids the internasal articulation only extends for a short distance ventrally, and the paired nasal plates each slope away from the dorsal suture to form an A-shape in transverse cross-section. There is a space enclosed between the two nasal plates, in the ventral vacuity of the A. The nasal plates of *Tsintaosaurus spinorhinus* are unusual in that they wrap around ventromedially (rostromedially in articulation, due to the dorsal rotation of the nasals) to meet again at the midline in a second internasal articulation at a thick ridge along much of the rostral surface of the tubular process (Figure 3D), forming a broadly O-shaped cross-section.

An alternate interpretation might be that the plates of the nasals are absent, and the tubular process of the nasals represents the upper portion of the A-shape described above. The vacuity in the nasals may then represent a subsequent invagination of the bones. We consider this less likely, but we cannot discount the possibility. In either case, the nasals of *Tsintaosaurus spinorhinus* are highly derived relative to those of other hadrosaurids.

IVPP V725 also exhibits an unusual apomorphic extension of the nasal bones into the intracranial space, producing a block-like structure that descends rostral to the braincase (Figures 2E and 3A, [17]: text fig. 2). That this structure is composed entirely of the nasals is shown by the Paratype skull, in which the nasals are not preserved and the block-like intracranial process is absent as well (Figure 4). In the holotype, the block tapers dorsally and slightly rostrally between the upturned margins of the frontals and prefrontals into the tubular process, and flares ventrally to span the space between the prefrontals. On the rostral face of the block are paired, dorsoventrally elongate, elliptical excavations bordering a thick, sagittal, dorsoventrally-oriented ridge (Figure 3A). The more complete right depression is emarginated by a thick rim laterally, and extends ventrally deep into the intracranial space on the face of a triangular lateral process. The ventral portion of the thickened septum between the depressions is broken, and it is unclear if it descended as deeply as the lateral process. Each depression extends dorsally up the nasal, and somewhat rostrally following the curve of the bone to a point just above the level of the skull table and just below the midline ridge on the tubular process, where it shallows to a transversely oriented rim. Between the depressions, the midline ridge also shallows and thins dorsally, to disappear at the same level as the depressions. 

#### Premaxilla

Young [17] referred the nearly complete rostral region of a premaxilla, IVPP K107, lacking the caudal extensions of the medial and lateral processes, to *Tsintaosaurus spinorhinus* (Figure 5). As detailed below, the morphological attributes of the oral margin and narial fossa of this element are unlike those of the premaxillae of any known hadrosaurid, but are similar to those of Saurolophinae. We follow Young [17] in referring this premaxilla to *T. spinorhinus* because, according to that author, this element was found in fairly close association with the type cranium and hypodigm of *T. spinorhinus*, all contained within a quarry a few meters long, wide, and deep. Additionally, as described below, the premaxilla exhibits derived characters of Lambeosaurinae not found in any saurolophine. 

The preserved rostral region of the premaxilla of *Tsintaosaurus spinorhinus* is subrectangular and three times longer than wide (Figure 5C). The degree of flaring of the rostral portion of this bone indicates that *Tsintaosaurus spinorhinus* had a relatively broad premaxillary ‘bill,’ unlike the narrow rostra of other lambeosaurines [8,9,13]. The caudoventral extreme or ‘corner’ of the bill is broadly rounded, as seen in saurolophines *Prosaurolophus maximus* and *Saurolophus* spp. [13], not sharply angled as in other lambeosaurines [8,9].

The oral margin, the relatively flat area dorsal and medial to the occlusal margin of the bone, is remarkably broad throughout its lateral extent, being nearly half as broad as the entire bone. The broad lateral oral margin of *T. spinorhinus* contrasts with the relatively thinner oral margin of the premaxilla of other lambeosaurines such as species of *Corythosaurus* [31,34], *Lambeosaurus* [8,35], *Parasaurolophus* [36,37], and *Hypacrosaurus* [38,39], and is more like that seen in saurolophines like *Edmontosaurus* [40] and *Brachylophosaurus* [41]. Rostrally, the convex oral margin of *T. spinorhinus* narrows abruptly to less than half of the maximum breadth that the oral margin achieves laterally; similar thinning of the oral margin rostromedially is observed in other lambeosaurines. The oral margin is clearly ‘reflected,’ strongly emarginated by the circumnarial fossa rostrally as in Saurolophinae, and this morphology extends along the lateral oral margin where it becomes less pronounced. The reflected oral margin is continuous with the folded margin of the caudolateral process of the premaxilla laterally (see below).

A faint emargination extends from the rostral, reflected oral margin across the rostrolateral oral margin of the premaxilla, and appears to represent the dorsalmost extent of the ramphotheca, forming the dorsal border of the beak attachment surface (Figure 5B, C, F). This ridge is apparently continuous with the dorsal extent of the oral margin medially, and gradually descends ventrally as it wraps around the bill, closely approaching the occlusal margin of the bone at the ventrolateral end of the premaxillary rostrum. Ventrally, the rostral margin of the occlusal surface of the premaxilla exhibits a series of six large, evenly spaced, subtriangular denticles (Figure 5C, E). 

The medial sagittal lamina, or prenarial septum (Figure 5B, C), of the premaxilla is continuous along the length of the fragmentary premaxilla and is not perforated by the apertura ossis nasi, indicating that the latter structure has been dorsally displaced, as in other Lambeosaurinae. Folds of the premaxilla extend from the dorsal and ventral margins of the bone to enclose the circumnarial fossa and form a secondary enclosure of the nasal vestibulum, here termed the pseudonaris, surrounded by the premaxilla. The pseudonaris (Figure 5B) is a neomorphic structure unknown outside of Lambeosaurinae, in which folds of the premaxilla enclose the distal portion of the nasal vestibulum, while the true apertura ossis nasi that conducts the vestibulum into the intracranial space proper is enclosed within the crest proper [42].

The lateral fold of the premaxilla is quite wide mediolaterally and remains broad as it curves rostrally to become the reflected oral margin (Figure 5B, C, F), unlike in other lambeosaurines where it thins abruptly before becoming the oral margin at the caudolateral ‘corner’ of the premaxillary bill. The dorsal fold is evidenced by a damaged ridge of bone in the caudal quarter of the element cutting across the accessory fossae of the circumnarial fossa (Figure 5D, F). Both folds converge posteriorly, but if they met they did so beyond the preserved limits of the bone. The pseudonaris they formed was thus elongate, as in *Corythosaurus* spp. [34] and not abbreviated and lacriform as in *Parasaurolophus walkeri* [36] and *Hypacrosaurus altispinus* [39].

Unlike the smooth and featureless circumnarial fossa typically present in other lambeosaurines [39], that of *Tsintaosaurus spinorhinus* is compartmented into three accessory fossae separated by two oblique ridges (Figure 5D). These ridges are inclined lateroventrally and their long axes are oriented rostrolaterally. The accessory fossae that are bounded by these ridges are narrow and deep. The rostral fossa is lacriform and entirely confined within the preserved margins of the bone. The median and caudal fossae are elongate and extend caudally into the narial passage, where the median fossa may have been truncated against the medial fold. Although morphologically somewhat different, compartmentalization of the narial fossa is commonly present in saurolophine hadrosaurids, including *Gryposaurus* [43,44], *Prosaurolophus* [45,46], *Edmontosaurus* [47,48], and *Brachylophosaurus* [49,50]. There is no indication in *T. spinorhinus* of the premaxillary foramen typical of saurolophines [13] and at least some non-hadrosaurid iguanodontians [51], but there are two regions of damage to the thin bone between the medial and lateral ridges that may have been perforated in life.

A different region of the premaxilla of *Tsintaosaurus spinorhinus* is preserved in IVPP V829. As noted above, this is a blocky subrectangular structure that surrounds and articulates laterally with the rhomboid distal process of the nasal (Figures 1 and 2). Given its dorsal position in the skull, indicated by the articulation with the distal process of the nasal, we interpret this fragment of premaxilla as the nearly complete right and partial left sides of the dorsal summit of the supracranial crest of *T. spinorhinus*. Most of the lateral surface of the premaxilla is excavated for reception of the distal nasal process (Figure 2C). In IVPP V829 the rhomboid nasal process is partially displaced ventrally from its articular position (Figure 2C and D), allowing observation of the morphology of the rostral and caudal margins of the lateral fossa. The contour described by those borders mirrors the rhomboid outline of the distal nasal process, indicating that the process fit tightly against the premaxilla. The rostral and caudal margins of the articular fossa for the nasal rise abruptly and prominently from the surrounding rostral region of the lateral surface of the premaxilla. The rostral margin is relatively broad, gently concave, and slightly recessed relative to the caudal margin and the rostral edge of the articular fossa. Adjacent to the articular fossa for the nasal, the eroded rostral region of the premaxillary fragment (where the bone separated from the rest of the premaxilla) is further compressed mediolaterally (Figure 1E, G), and forms a solid extension of the crest beyond the hollow nasal passages, or ‘cockscomb.’

The ventral surface of IVPP V829 is expanded mediolaterally and strongly concave. This concavity is D-shaped in ventral view and occupies the rostral half of the ventral surface of the bone fragment (Figure 1E, F). It exhibits a pair of narrow vascular grooves that are parallel and longitudinally arranged, one on either side of the sagittal plane of the crest. Dorsal and medial to the ventral concave surface of the premaxillary fragment, there is a large foramen that opens rostromedially (Figure 1D). At the caudal region of the premaxillary fragment, the ventral concavity is continuous with a caudoventrally-directed, hook-like process (Figure 1D, E). Although only the right caudoventral process is preserved, the bilateral symmetry of the premaxillae suggests that most likely a left process was also present. In addition to its hook-like caudoventral curvature, the process is also slightly curved medially. The lateral edge of the process is continuous with and forms a 125° angle with the lateral border of the rostral D-shaped ventral concavity. The ventral surface of the process is slightly concave and smooth. In ventral view, its elongated contour, with the slightly wider caudal end, is that of a narrow paddle. A median space lies in between the hook-like processes and deepens dorsally, adjacent to the caudal end of the D-shaped ventral concavity of the bone. This space is divided by a short longitudinal ridge that is coincident with the sagittal plane of the cranial crest. Caudal to the hook-like process, the ventral surface of the fragment is shallowly concave and evidently formed a small secondary chamber (Figure 1F).

A small, triangular bony fragment partially overlaps the dorsolateral surface of the right hook-like caudoventral process of IVPP V829, just ventral and adjacent to the proximoventral margin of the partially disarticulated rhomboid nasal process (Figure 1C, E). This fragment represents a different element than the nasal and premaxillary fragments described above. In the absence of data indicating otherwise and given its position in IVPP V829, we tentatively suggest that it represents the caudodorsal extreme and only preserved portion of the lateral process of the premaxilla. 

#### Prefrontal

The prefrontal of *Tsintaosaurus spinorhinus* is unique in possessing a rostral process that projects significantly dorsally (Figure 6; this bone was identified in IVPP V818 as part of the nasal by Young [17]). This rostral ascending process projects dorsally and twists medially along its mid-length, so that the external surface of its dorsal segment faces laterally (Figure 6A, B). The dorsal extremity is mediolaterally compressed and its narrow dorsal margin is inscribed with short transverse indentations (Figure 6A). When the prefrontal is articulated on the lateral side of the skull, the ascending process buttresses the base of the nasal, as previously noted by Young [17] in IVPP V725 and Buffetaut and Tong-Buffetaut [25] in IVPP V818. Another unique trait of the prefrontal of *T. spinorhinus* is a thin flange that extends laterally from the rostral margin of the lacrimal process to the proximal region of the ascending process (Figure 6A, B). The caudodorsal external surface of this flange is concave and smooth, and continuous dorsomedially with the proximal region of the ascending process. The medial surface of the main body of the prefrontal shows a deeply indented articular surface for the frontal. Long longitudinal grooves excavate the ventral portion of the ascending process and the main body of the prefrontal (Figure 6D). Caudally, the central body of the prefrontal is continuous with a deep postorbital process.

#### Frontal

Our observations in IVPP V725 and V818 confirm those of Young [17] and Buffetaut and Tong-Buffetaut [25] regarding the dorsal curvature of the rostral region of the ectocranial surface of the frontals. This stands in contrast to most lambeosaurins, in which the frontal is down-warped as part of the platform supporting the crest. The frontals form a narrow process between the nasals rostrally, as in *Jaxartosaurus aralensis* [15] and *Kazaklambia convincens* [52], but in *Tsintaosaurus spinorhinus* this process clearly ascended to participate in the crest as reconstructed by Young [17].

The Paratype skull, IVPP 818, is missing the nasal, and reveals details of the nasofrontal articulation. The rostral surface of the frontal is deeply embayed along the sagittal plane endocranially, and features a weakly-striated, tongue-like process extending rostrally from the rostrolateral margin, above which is a nearly smooth, concave surface for articulation with the nasals (Figure 4). The ectocranial surface of the frontal forms a shelf that overhangs this articular surface and the tongue-like process, extending rostrodorsally well rostral to the tongue-like process and concealing it in dorsal view (Figure 4). The concave area dorsal to each tongue-like process forms a slot or socket into which the nasal must have fit, effectively clamping it between the tongue-like process ventrally and the ectocranial process dorsally.

The tongue-like processes of the nasofrontal articulation are not continuous across the midline of the skull; they taper into the embayed rostral margin of the bone before reaching the sagittal plane. The overlying ectocranial process is however continuous across the midline of the skull. The articular socket dorsal to the tongue-like process extends laterally and is divided from a dorsoventrally deep beveled facet for the prefrontal by a thick, dorsoventrally oriented ridge. There is a small notch at the base of this ridge where the lacrimal process of the prefrontal apparently clips between the ventral tongue-like process and ectocranial process of the frontal. This ridge, at the edge of the ectocranial process of the frontal, buttresses the caudal margin of the prefrontal for some distance dorsally before the nasal interposes between them.

## Discussion

### Elevation of the Nasals in *Tsintaosaurus spinorhinus*

We support Young [17] and Buffetaut and Tong-Buffetaut [25] in their contention that the nasals of *Tsintaosaurus spinorhinus* projected dorsally from the rostral region of the skull roof. The ectocranial surface of the frontal is clearly inflected dorsally in both the type (Figure 3) and Paratype (Figure 4) specimens. The distinct ascending process of the prefrontal (present in both the Paratype specimen IVPP V818 and the referred, isolated prefrontal IVPP V725, Figure 6) and its dorsal articulation with the nasals also supports this conclusion. Hadrosaurid skulls are typically crushed either mediolaterally, dorsoventrally, or obliquely; we know of no case in which the skull roof is bent through a right angle as proposed for specimens of *Tsintaosaurus*. It seems unlikely that *post mortem* distortion would act in exactly the same atypical manner on both the type and Paratype skulls, warping the frontals, prefrontals, and in IVPP V725 the nasals to a dorsal orientation. Further, the block-like intracranial process of the nasals (Figure 3) fits in the space rostral to the braincase when the nasal is elevated, but any rotation of the nasal to the horizontal around a fulcrum on the skull table (as implied by Taquet [24]) would no doubt bring this process into the rostral region of the braincase proper, where there does not appear to be enough space to accommodate it. In short, we reject the conclusion of Taquet [24] that the elevation of the nasals in *Tsintaosaurus spinorhinus* is anything other than the original configuration of the elements.

### The Crest of *Tsintaosaurus spinorhinus*

The above observations on the morphology and articular relationships of the prefrontal, frontal, nasal, and premaxilla of *Tsintaosaurus spinorhinus* led to a new reconstruction of the supracranial crest of this lambeosaurine. The iconic spike-like ornamental structure of *T. spinorhinus* hitherto depicted in popular dinosaur books [53–55] and various skeletal mounts and reconstructions [1,17,23,25] is in fact only part of a more elaborate crest structure. As in other lambeosaurines, the crest of *T. spinorhinus* was primarily composed of the premaxillae and nasals, with supporting contributions from the prefrontals and frontals. The model that emerges from combining the tubular process composed of the fused paired nasals (IVPP V725), with the expanded rhomboid distal process of the nasals (IVPP V725 and V829), and the blocky premaxillary fragment (IVPP V829) is that of a tall, lobate crest (Figure 7). 

In hadrosaurids, the premaxilla typically bifurcates caudodorsally into medial and lateral processes [1]. In lambeosaurines these two processes extend farther caudally and dorsally, and fold laterally to meet lateral to and enclose an internal space including the apertura ossis nasi [3,48,56,57]. The caudodorsal position of IVPP V829 that results from assembling this fragment with the tubular and partial rhomboid distal process of the IVPP V725 nasal, indicates that V829 corresponds to the dorsal-most region of the medial process of the premaxilla, as depicted in Figure 7. This interpretation is consistent with the presence of the sagittal plane within the element, and the fact that, caudal to the pseudonaris, the medial process extends dorsally relative to the lateral process in all known lambeosaurines for which the caudal portion of the premaxilla is available [13]. Likewise, as discussed above, the small triangular bony fragment attached to the rostroventral margin of the rhomboid nasal process of IVPP V829 (Figure 2C, D) appears to represent the dorsal extent of the lateral process of the premaxilla. According to this interpretation, the lateral process of the premaxilla of *T. spinorhinus* would reach the base of the rhomboid process but remain ventral to the dorsalmost extent of the larger medial process. A similar configuration, albeit with different morphology, of the premaxillary lateral process relative to the premaxillary medial process and the nasal is typically present in ‘helmet-crested’ lambeosaurins (sensu Prieto-Márquez et al. [32]) like *Corythosaurus* [31,34], *Lambeosaurus* [8,35], and *Hypacrosaurus* [39,58]. Articulation of the premaxillary lateral process with the nasals may have occurred along the longitudinal sagittal ridge on the rostral surface of the fused nasals (Figure 3D), at approximately mid-length of the tubular process (Figure 7B).

The great height of the crest of *Tsintaosaurus spinorhinus*, which exceeds the depth of the skull along the long axis of the quadrate, is revealed by the length of the nasals above the skull roof. In our model, the tubular process of the nasal projects dorsally at approximately 90° from the horizontal plane of the skull roof, rather than rostrodorsally as in previous reconstructions [1,17,23]. The apparent rostrodorsal inclination of the tubular process of the nasals is due to postdepositional deformation of these elements. This is indicated by the presence of an oblique fracture at mid-length of the processes and subsequent slight rostral displacement (see [17]: text fig. 2). This erect crest is then angled somewhat posteriorly due to the caudoventral inclination of the caudal region of the skull roof. Caudoventral sloping of the skull roof is indicated by articulation of all available elements of the skull (Figure 7B) and is typically present in lambeosaurines ([13], character 192).

Because in lambeosaurines the rostrodorsal margin of the prefrontal commonly underlies the lateral process of the premaxilla [2], we may assume, in the absence of data indicating otherwise, the same relationship between these elements in *Tsintaosaurus spinorhinus*. However, in the *T. spinorhinus* the prefrontal displays an autapomorphic flange between the lacrimal and ascending processes. The incompletely preserved flange of IVPP V725 extends laterally from the area above and rostral to the orbit. Following the assumption that this flange, given its rostrodorsal location in the prefrontal, probably met the lateral process of the premaxilla, we hypothesize that it must have curved rostrally at some point along its width. It is not certain whether in that area the lateral process of the premaxilla also extended laterally and then curved caudally to meet the prefrontal in that area. However, the curvature of the prefrontal alone would likely create a bulging area or ‘swelling’ on the lateral surface of the facial skeleton of *T. spinorhinus*, just above and rostral to the orbit (Figure 7).

#### Internal morphology of the crest

Because of the limited material of the crest, we are only able to reconstruct some aspects of the internal anatomy of the crest and the path of the nasal passage (Figure 8). Lambeosaurines are characterized by enclosure of the nasal vestibule by the premaxillae [8]. It is unclear if the rostral portion of the vestibule was completely enclosed in *Tsintaosaurus spinorhinus*, because of the incomplete nature of the premaxilla. However, as noted above, the outgrowths of the premaxilla that define the pseudonaris appear to converge caudally, suggesting that the vestibule was indeed enclosed by bone before reaching the crest. We hypothesize that the posterior portions of the vestibule (within the crest proper) were also enclosed, but lacking a complete premaxilla we cannot be certain.

As noted above, the premaxillary fragment represented in IVPP V829 shows a concave ventral surface. Its hypothetical relatively high position within the crest of *Tsintaosaurus spinirhinus*, just below the level of the rhomboid distal process of the nasal (Figure 7B), suggests that it probably constitutes the roof of the common median chamber [3]. The common median chamber is an osteologically undivided supraorbital extension of the nasal cavity that has been documented in lambeosaurine genera like *Corythosaurus*, *Lambeosaurus*, *Hypacrosaurus*, and *Parasaurolophus* [9,34,38,39]. The common median chamber is enclosed by variable contributions of the nasals and premaxillae that form the hollow supracranial crests of these animals [59]. 

The hook-like medial processes of the premaxilla appear to divide the common median chamber into two, unequal portions. The larger, rostral chamber is less complete; it was bounded dorsally and anteriorly by the medial process of the premaxilla, and the lateral processes may have covered it at least partially laterally. This was most likely where the osseous nasal ducts from the external naris conducted air into the crest cavity proper. Because of incomplete preservation, we cannot reconstruct the extent of the lateral diverticula, nor their relationship to the common median chamber.

The hook-like processes of the premaxillae form the lateral margins of a substantial midline foramen within the crest passing between the sub-chambers. The smaller caudal chamber is bounded dorsally and caudodorsally by the medial process of the premaxilla, caudally by the nasals, and laterally by the premaxilla. Air presumably passed from the rostral to the caudal chamber through the interconnecting foramen before being directed ventrally into the cranium proper.

We do not believe that the nasal passage passed through the hollow tubular process formed by the nasals (*contra* [17]). Not only would this be unprecedented in hadrosaurid anatomy, but the cross-sectional area of the tubular process of the nasals is quite small and there is no ventral exit to the tubular process by which the enclosed tube could communicate with the intracranial space. 

We reconstruct the nasal passage as passing ventrally down the rostral face of the nasal bones to enter the cranial cavity rostral to the orbits. Typically, a large foramen at the ventral limit of the common median chamber communicates between the latter and the antorbital space [2,42]. This foramen is not preserved in *Tsintaosaurus spinorhinus*; however, the entrance of the nasal passage into the intracranial space is marked by the two large elliptical depressions on the block-like intracranial process of the nasal described above (Figure 3A). These depressions would have bounded the nasal passage caudally, whereas the premaxilla and possibly the prefrontal would form its rostral and lateral walls. Note that the previously described swelling of the lateral surface of the cranium occurs approximately at this level, and it may be a structural consequence of threading both the dorsal and ventral nasal duct through the otherwise relatively narrow base of the crest. Indeed, it appears that the crest was mediolaterally quite broad rostrodorsal to the orbits. 

It may be significant that the entrances of the nasal passage into the intracranial space are apparently paired. The foramen communicating between the crest and the cranial cavity [2,52] is undivided in other lambeosaurines. Our interpretation suggests that, in *Tsintaosaurus spinorhinus*, airflow was separate on left and right sides of the crest after passing through the common median chamber. The simplest interpretation would be that airflow from each naris was completely separate through the entire crest, and did not mix in the common median chamber. The latter may have therefore been divided by an unossified sagittal septum, possibly extending from the sagittal ridge on the dorsal surface of the common median chamber noted above.

#### Function of the tubular process of the nasal

As noted above, we doubt that the nasal tubular process, if indeed it is hollow, transmitted the nasal passages into the intracranial space. We have reconstructed this area as housing a diverticulum of the crest cavity (Figure 8). However, the unusual shape of the nasals warrants further mention. A hollow cylinder is structurally stronger than a solid one, and we suspect that the apomorphic structure of the nasals may be an adaptation to support the weight of the crest. In other lambeosaurines, the crest rests on the dorsal surface of the frontals and prefrontals, and its weight is transferred to the skull roof ventrally via these bones. In *Tsintaosaurus spinorhinus*, the ectocranial connection to the frontal is limited to edge-on contact. Presumably the intracranial process of the nasals slotted into the peculiar nasofrontal articulation caudally, where reciprocal processes of the nasal would have been ‘clipped’ in between the tongue-like processes and the overlying concave articular surface.

We suspect that the weight of the crest was transferred via the complex socketed arrangement of the rhomboid processes to the tubular process of the nasals, and then ventrally to the prefrontals and the block-like intracranial processes of the nasals, where it was then passed to the skull roof via the tongue-like rostral processes of the frontal. In this regard, it may be significant that the prefrontals do not underlie the crest as part of the frontal platform, but buttress the crest laterally. In other lambeosaurines, the extension of the frontal platform onto the prefrontal (see below) may have permitted greater support of the crest laterally, relieving the nasals of more of the burden of the crest, and explaining the absence of a tubular nasal process in these taxa.

### The Nasofrontal Articulation and Evolution of the Lambeosaurine Frontal Platform

**Figure 9:**
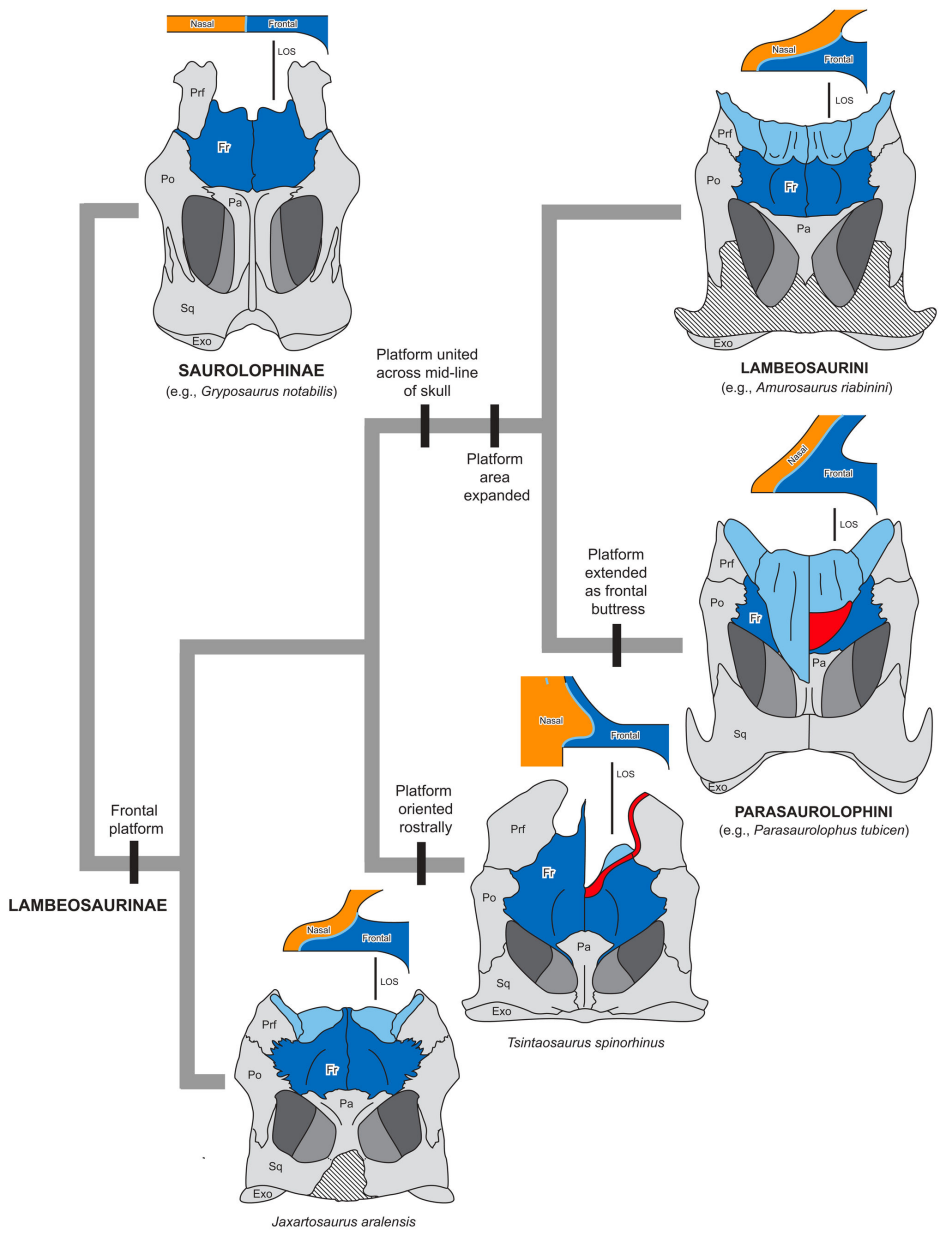
Nasofrontal articulation of selected hadrosaurids in phylogenetic context. For each terminal taxon, the lower figure is a reconstruction of the skull roof in dorsal view and the upper figure is a schematic cross-section along the line of section (LOS) indicated. The frontal has been highlighted in dark blue for clarity, the nasal is orange in cross-sections (and omitted from dorsal view for clarity), and the nasofrontal/nasoprefrontal articulation surface is in light blue. Cut bone is red and missing bone is cross-hatched. *Gryposaurus notabilis*, AMNH 5350; *Tsintaosaurus spinorhinus*, combination of IVPP 725 and 818; *Jaxartosaurus aralensis*, PIN (Paleontological Institute, Moscow, Russia) 1/5009; *Amurosaurus riabinini*, AENM (Amur Natural History Museum, Blagoveschensk, Russia) 1/232; and *Parasaurolophus tubicen*, PMU (Paleontological Museum, Uppsala, Sweden) R222.

**Figure 10:**
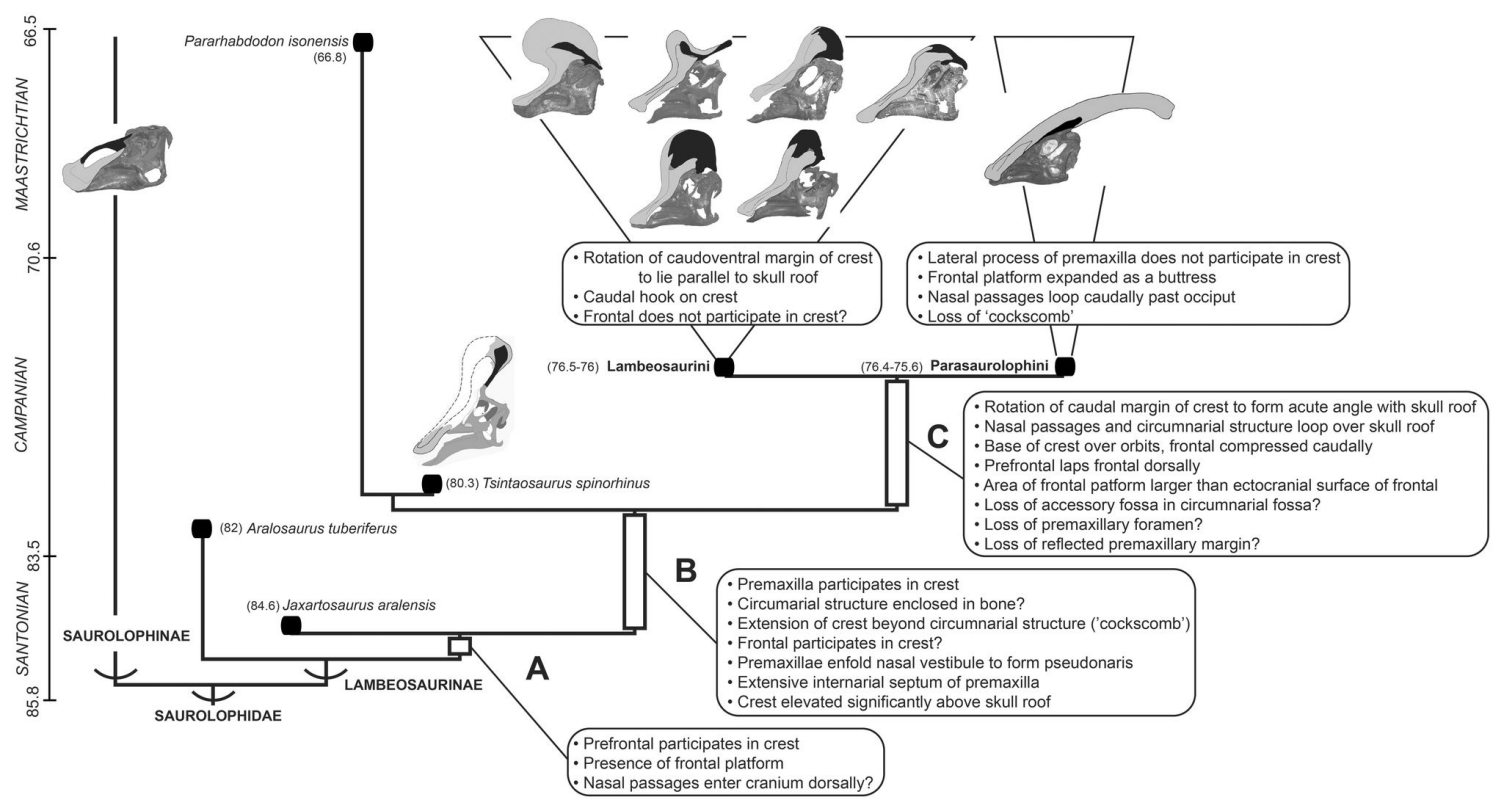
Simplified time-calibrated phylogram of Lambeosaurinae. Topology based on [14], with characters discussed in text optimized. In each hadrosaurid skull, light grey indicates premaxilla and black denotes nasal. The literature sources for each of the taxon’s datings are as follows: *Aralosaurus tuberiferus* [60,61], *Jaxartosaurus aralensis* [62], *Pararhabdodon isonensis* [21], *Tsintaosaurus spinorhinus* [33], and Lambeosaurini (oldest recorded age being that of *Corythosaurus casuarius*) and Parasaurolophini (oldest recorded age being that of *Parasaurolophus walkeri*) [63]. Geochronological ages are from [64].

In lambeosaurin hadrosaurids, the nasal articulation of the frontal and prefrontal form a wedge-shaped support structure for the overlying nasal, a configuration termed ‘frontal platform’ [13]. This platform is typically bounded caudally by a raised rim of the frontal, and laterally by the dorsomedial flange (or ‘crest’) of the prefrontal, forming a dorsally convex cradle for the nasals. Despite the differences, the articulation in *Tsintaosaurus spinorhinus* shares a wedge-shaped portion underlying the nasal, and a raised caudal rim defining the edge of a concave support area for the nasal with the morphology typically identified as a frontal platform. The tongue-like processes form the wedge, and the overhanging ectocranial process of the frontal may be considered a hypertrophied version of the raised caudal margin (Figure 9). The nasofrontal articulation in *Tsintaosaurus spinorhinus* corresponds topologically, materially, structurally, and functionally with the conventional frontal platform. Because a frontal platform is also present in *Jaxartosaurus aralensis* [15], optimization on the lambeosaurine phylogeny of Prieto-Márquez et al. [14] (Figures 9 and 10) suggests that a platform may be ancestral for *T. spinorhinus*.

There is, therefore, every reason to suspect that the nasofrontal articulation in *T. spinorhinus* is homologous to the frontal platform of other lambeosaurines. The obvious differences between this structure and a conventional frontal platform can be explained as consequences of the fact that the nasal is positioned rostral to the frontal, and not dorsal to it as in most lambeosaurines. As such, we would expect the nasofrontal articulation to be oriented rostrally. This is indeed the case in *Tsintaosaurus spinorhinus*, in which the nasofrontal articular surface is not visible in dorsal view (Figure 4).

This nascent frontal platform observed in *Tsintaosaurus spinorhinus* is very short rostrocaudally, shorter than the more conventional platform of *Jaxartosaurus aralensis* [15]. As in the latter taxon, this structure is not continuous across the midline of the skull, instead being isolated laterally as two distinct, paired surfaces, with a hollow space along the midline of the skull that was occupied by the intracranial process of the nasal in life. A rostral embayment in the frontal is a common feature of hadrosaurids [13], but in this case it is significant in that it completely interrupts the frontal platform medially.

The frontal forms a platform with limited dorsal area (smaller than the area of the ectocranial surface of the frontal) in both *Jaxartosaurus aralensis* and *Tsintaosaurus spinorhinus* (in which it is concealed from dorsal view by the overhanging ectocranial process of the frontal), and is split into lateral lobes by the intervening ectocranial surface of the frontals in both taxa. The manner of the split is different in these taxa, however, as in *J. aralensis* the medial extension of the frontal does not appear to participate in the crest, whereas it certainly does in *T. spinorhinus*. In later lambeosaurines the frontal platform has greater dorsal area than the frontal, and the split in the platform is manifested as a cleft in the frontal articular surface (Lambeosaurini, sensu Prieto-Márquez et al. [32]), or is absent entirely (Parasaurolophini, sensu Prieto-Márquez et al. [32]). It is unclear whether the fully split frontal platform is ancestral for Lambeosaurinae or convergent in *J. aralensis* and *T. spinorhius*, but the presence of a platform appears to be ancestral for the group. This further suggests that the nominal ‘loss’ of the platform in Parasaurolophini appears to be achieved by transformation of that structure into an extended frontal buttress through dorsal extension and elaboration of the caudal rim of the frontal (Figure 9).

### Early Evolution of the Cranial Crest in Lambeosaurinae

The premaxilla and nasal bones experienced extreme morphological transformations during lambeosaurine evolution (Figures 9 and 10). In non-lambeosaurine hadrosaurids, such as saurolophine hadrosaurids and outgroup taxa to Saurolophidae, the nasal typically constitutes the dorsal surface of the rostrum anterior to the orbit [1,4]. In contrast, in lambeosaurine hadrosaurids the premaxilla and nasal migrated caudally and dorsally as part of a rotation of the facial skeleton and partial transposition of elements of the rostrum to the skull roof [56,57]. The new interpretation of the crest of *Tsintaosaurus spinorhinus* offered here, and this species’ intermediate phylogenetic position relative to better-known Parasaurolophini and Lambeosaurini permit an exploration of the sequence of acquisition of characteristic lambeosaurine traits. Atomization of the complex transformation of the lambeosaurine facial skeleton into individual characters allows for their optimization on the phylogeny (Figure 10). To some extent, this is limited, both by the fragmentary nature of the three principle stem-lambeosaurine taxa involved, *A. tuberiferus*, *J. aralensis*, and *T. spinorhinus*, and by uncertainties regarding the ancestral states of some characters. However, certain transitions can now be traced with some clarity.

*Aralosaurus tuberiferus* already possesses a crest [19], and indeed, that is likely the ancestral state for Saurolophidae [57]. However, in *A. tuberiferus* the nasal passages apparently still entered the cranium anteriorly as in ancestral hadrosaurids. This entrance rotated such that the nasal passages enter the cranium dorsally in the lineage leading to the clade consisting of *T. spinorhinus*, Lambeosaurini, Parasaurolophini, and their most recent common ancestor (Figure 10, branch B), probably before the divergence of *J. aralensis* (Figure 10, branch A). Because of specimen incompleteness, we cannot determine the branch on which the crest was elevated to a significant distance above the skull table, but the latest this occurred was on branch B (Figure 10). These transitions evidently occurred prior to the rotation of the caudal margin of the crest to an acute angle with the skull table (‘folding’ of the nasal), and before the base of the crest was shifted caudally to the region directly dorsal to the orbits and the nasal passages were looped over the skull roof on branch C (Figure 10).

In other lambeosaurines, the medial process of the premaxilla forms part of the crest, and this is true of *Tsintaosaurus spinorhinus*. Unfortunately, partial preservation of other lambeosaurines only permits the conclusion that the medial process of the premaxilla was incorporated into the crest by branch B. (Figure 10). In Lambeosaurini, the distal end of the lateral process of the premaxilla also forms a part of the crest (e.g., *Corythosaurus* spp. [31,34], *Hypacrosaurus* spp. [39]), while this process appears to be restricted to the rostrum in Parasaurolophini [1, 37]). If we have correctly interpreted the presence of the lateral process of the premaxilla on the crest of *T. spinorhinus*, then participation of this element in the crest may have been present ancestrally, again by branch B, and its absence in Parasaurolophini would be a reversal. Otherwise this is an independent acquisition in *T. spinorhinus* and Lambeosaurini, and at least in the latter case it must have evolved after participation of the medial process in the crest. However, further optimization of this character is ambiguous because no premaxilla is known for *Ararlosaurus tuberiferus* [19] and *Jaxartosaurus aralensis* [16].

In Lambeosaurini and Parasaurolophini, folds of the premaxillary processes wrap around the outside of the crest, enclosing the circumnarial structure and nasal passages and forming part of the ‘hollow crest’ structure [56,57]. While no complete premaxilla is known for *Tsintaosaurus spinorhinus*, IVPP V829 preserves, as indicated above, segments of the medial and possibly lateral processes (Figure 1C, E). We cannot be certain to what extent these processes enclosed the space within the crest, whether there were fontanelles [39] or not, but we can be certain that the crest space was at least partially enclosed along branch B (Figure 10).

The nasals of lambeosaurins and parasaurolophins also possess lateral processes that fold over the lateral surface of the crest to enclose part of the hollow space within. In contrast, the nasals of *Tsintaosaurus spinorhinus* appear to lack lateral processes, and the nasals do not house internally any significant portion of the space within the crest, although they participate in the posterior margin of the common median chamber (see above). It is not clear if the lateral processes are absent, or if the tubular process of the nasals itself is composed of these lateral processes in *T. spinorhinus*; the tubular structure of the nasals of *T. spinorhinus* is so unusual for a hadrosaurid that it is difficult to draw reliable conclusions about homology. If the absence of these processes does represent the ancestral state for Lambeosaurinae, that would indicate that enclosure of the crest may have been initially accomplished by the premaxillae alone. Alternately, loss of these processes may be apomorphic for *T. spinorhinus*.

*Tsintaosaurus spinorhinus* informs as to the sequence of incorporation of additional skeletal elements onto the crest. In Lambeosaurini, Parasaurolophini, *Jaxartosaurus aralensis*, and *Tsintaosaurus spinorhinus* the prefrontal participates in the crest to some degree, indicating that it was incorporated on branch A (Figure 10). The prefrontal contribution to the crest in Lambeosaurini and Parsaurolophini is in the form of a thin ascending mediodorsal flange; this flange is present in *J. aralensis* [15], and we regard the rostral ascending process of the prefrontal of *T. spinorhinus* as a possible hypertrophied homolog of that structure. In *T. spinorhinus* the prefrontal does not participate directly in the frontal platform, and this is likely a reversal, although participation of the prefrontal in the frontal platform may be convergent in *J. aralensis* and Lambeosaurini + Parsaurolophini. In *T. spinorhinus* and *J. aralensis*, the prefrontal does not dorsally lap the frontal caudally, as in Lambeosaurini and Parsaurolophini, and this apparently evolved on branch C of the phylogeny (Figure 10).

In *Tsintaosaurus spinorhinus* and Parasaurolophini the frontal departs the profile of the skull roof to participate in the crest proper; this may be convergent, or alternately this evolved on branch B and was lost in Lambeosaurini (as reconstructed on Figure 10). In either case, incorporation of the prefrontal precedes incorporation of the frontal into the crest.

In *Tsintaosaurus spinorhinus* there is a solid extension of the crest beyond the margins of the nasal passages/circumnarial fossa. This ‘cockscomb’ has been previously considered a synapomorphy of Lambeosaurini (as part of the ‘helmet-shaped crest’ [13]). While it may be convergent in *T. spinorhinus*, we are inclined to interpret its absence in Parasaurolophini as a reversal. In either case, presence of a cockscomb cannot be considered to diagnose Lambeosaurini exclusively.

That elevation of the crest significantly above the skull roof precedes the displacement of the crest onto the skull roof proper and rotation of the caudal margin of the crest to form an acute angle with the skull roof may be significant. This pattern is also evident among Saurolophini (Prieto-Márquez et al., submitted.), in which the elevation of the crest preceded its displacement caudally over the orbits; these taxa also show a progressive incorporation of additional skeletal elements into the crest, as hypothesized here in lambeosaurines. Interestingly, certain transitions occur in the opposite manner in lambeosaurin ontogeny, with rotation of the caudal margin of the crest and displacement of the base of the crest onto the skull roof being present earlier in ontogeny than elevation of the crest [39: fig. 13]. We suspect that this may possibly be related to the ethological function of the crest, with a high crest signaling maturity.

Certain other characters of the caudal region of the skull considered morphogenetically or mechanically associated with development of the crest and its retraction over the skull roof in Saurolophini (Prieto-Márquez et al., submitted), including curvature of the quadrate and narrowing of the dorsal margin of the infratemporal fenestra, are present in *Tsintaosaurus spinorhinus*, alongside caudoventral inclination of the skull roof and down-warping of the sagittal crest on the parietal. If these characters are associated with crest development, they must be linked to elevation of the crest, since the crest is not retracted over the skull roof in *T. spinorhinus*. *Tsintaosaurus* lacks a strong sagittal/ nuchal crest extending dorsal to the skull roof, and this may then be related to the retraction of the crest.

Lambeosaurins are characterized as having helmet- or fan- shaped crests, while Parasaurolophins have tubular crests, and it is unclear which, if either, was the ancestral state for lambeosaurines. While the crest of *Tsintosaurus spinorhinus* is incompletely known, it suggests that there is more to the history of the lambeosauine crest than this simple dichotomy. As noted above, *T. spinorhinus* has a ‘cockscomb,’ as in Lambeosaurini. However, the cross-section of the crest must have been fairly broad relative to its rostrocaudal length, as in Parasaurolophini. We suspect that the typology of lambeosaurin and parasaurolophin crests may be insufficient for describing the ancestral lambeosaurine crest, which may have combined characteristics of both “types.” However, we must emphasize that the crest of *T. spinorhinus* may be highly derived, and it should not itself be shoe-horned into serving as a model for the early lambeosaurine crest.

In *Tsintaosaurus spinorhinus* the circumnarial structure is surrounded by excrescences of the crest bones (a ‘hollow crest’), the crest is already somewhat retracted, and the premaxilla is already incorporated into the crest. It is likely that these transformations occurred at different points in evolution, and the order in which they occurred might be significant. We predict that taxa possessing intermediate stages of evolution of the crest, such as a laterally ‘open’ crest, or nasal passages entering the skull rostrodorsally, remain to be discovered.

### Evolution of the Rostrum in Lambeosaurinae

The phylogenetic position of *Tsintaosaurus spinorhinus* also offers insight into the evolution of the lambeosaurine rostrum. In lambeosaurins and parasaurolophins, excrescences of the premaxilla fold over the nasal passage rostrally to form an osseous pseudonaris, the homologous apertura ossis nasi is dorsally displaced and enclosed within the crest [57], and the premaxillary prenarial septum is substantially longer than in saurolophine hadrosaurids. *T. spinorhinus* exhibits enfolding of the nasal passage and the prenarial septum extends for the length of the preserved fragment of the rostrum. This indicates that at least partial enclosure of the nasal vestibule occurred by branch B on the phylogeny (Figure 10), as did dorsal displacement of the true apertura ossis nasi, and these changes preceded the rotation of the crest to an acute angle with the skull roof. Indeed, in lambeosauin ontogeny, at least partial enfolding of the pseudonaris appears to be present at a very early stage, prior to differentiation of the crest itself (e.g., 39).

The rostrum of other lambeosaurines is relatively featureless and unexpanded, lacking a broad ‘bill,’ a reflected premaxillary ‘lip,’ accessory fossae within the circumnarial fossa, and a premaxillary foramen. This configuration has been tacitly accepted as reflecting retention of ancestral characters, as these features are generally considered absent in non-saurolophid (sensu Prieto-Márquez [13]) iguanodontians [13]. However, *Tsintaosaurus spinorhinus* displays a thick, reflected occlusal margin of the premaxilla more similar to that seen in Saurolophinae than in other Lambeosaurinae. *T*. *spinorhinus* also exhibits complex subdivision of the circumnarial fossa and it is uncertain whether a premaxillary foramen is present as well. While these characters might be convergent between *T. spinorhinus* and Saurolophinae, we suspect that they indicate that lambeosaurines ancestrally possessed a more conventional, morphologically complex, Saurolophine-like rostrum. Certainly, it seems unparsimonious to accept that all of these similarities developed convergently, independent of one another.

Many aspects of the ‘complex rostrum’ of Saurolophinae and *T. spinorhinus* are anatomically related to the circumnarial fossa, and their loss in Lambeosaurini and Parasaurolophini has been suggested to be a consequence of displacement of the narial apparatus and circumnarial structure to the roof of the skull, and/or to enclosure of the circumnarial fossa by the premaxilla [57]. However, the association of a complex rostrum with an enfolded premaxilla and a retracted apertura ossis nasi argues for some degree of independence in these characters. If the ‘complex rostrum’ is indeed ancestral, then its presence in *Tsintaosaurus* suggests that the ‘simplification’ of the lambeosaurine rostrum occurred after enclosure of the nasal vestibulum by the premaxilla and after retraction of the apertura ossis nasi, on branch C of the phylogeny presented in Figure 10.

## Methods

The osteological descriptions and observations presented in this study are based on examination of the holotype specimen and referred materials of *Tsintaosaurus spinorhinus* housed at the IVPP (Beijing, China). Permission was granted to APM to access the IVPP collections and examine *T. spinorhinus* fossils. None of the specimens were purchased, donated, or loaned; the specimens were observed in situ at the IVPP collections.
